# Feasibility and safety of “filter-thrombus integrated retrieval technique” based on controlled deformation of spindle shaped IVC filters for complex IVC filter retrieval

**DOI:** 10.3389/fradi.2026.1774148

**Published:** 2026-03-05

**Authors:** Zexiang Wu, Gang Chen, Wenming Wang, Yingjiang Xu, Zhu Wang, JianWei Gao, Yang Geng, Jiahao Zhu

**Affiliations:** Department of Interventional Vascular Surgery, Binzhou Medical University Hospital, Binzhou Binzhou Medical University, Binzhou, Shandong, China

**Keywords:** endovascular technique, filter retrieva, filter thrombosis, inferior vena cava filte, pulmonary embolism, venous thromboembolism

## Abstract

**Objective:**

To evaluate the safety and feasibility of the “filter-thrombus integrated retrieval technique,” which leverages the controllable deformation characteristics of spindle-shaped inferior vena cava (IVC) filters, for the retrieval of filters with thrombus burden.

**Methods:**

A retrospective analysis was conducted on clinical data from 51 patients with IVC filter thrombosis (IVCFT) treated at our center from September 2023 to September 2025. All patients underwent retrieval using the “filter-thrombus integrated retrieval technique” to construct a “filter-thrombus complex” for *en bloc* removal. Preoperative 3D-DSA was employed to quantitatively assess thrombus volume. The primary endpoint was the technical success rate. Secondary endpoints included the incidence of pulmonary embolism (PE) and procedure-related complications.

**Results:**

The mean filter dwell time was 35.25 ± 5.80 days. 3D measurements revealed a mean thrombus volume of 4.60 ± 0.42 cm^3^, with 13 cases classified as severe thrombus burden. The technical success rate was 100% (51/51). No symptomatic PE occurred postoperatively. Routine CTPA screening detected asymptomatic, minor PE in 4 patients (7.8%), all of which resolved following anticoagulation. No serious complications, such as venous avulsion or major hemorrhage, were observed.

**Conclusion:**

The filter-thrombus integrated retrieval technique appears to be a safe and feasible strategy for managing thrombosed filters. Through a unique physical “caging” mechanism, this technique minimizes the risk of iatrogenic PE during retrieval without relying on additional thrombolysis or aspiration devices. It presents a viable option for patients with high thrombus burden or contraindications to anticoagulation.

## Introduction

Retrievable inferior vena cava (IVC) filters are devices deployed within the IVC to intercept thromboemboli originating from the deep veins of the lower extremities, thereby preventing fatal pulmonary embolism (PE). These devices provide mechanical prophylaxis against PE and are intended for safe retrieval once the risk of thromboembolism has subsided. However, clinical data indicate that the actual retrieval rate of these devices is as low as 33% (1). Prolonged filter retention not only escalates medical costs but also significantly increases the risk of complications, including filter-associated thrombosis, fracture, migration, and vessel wall penetration. Therefore, timely and safe retrieval is a critical component in optimizing the clinical utility of IVC filters.

Inferior vena cava filter thrombosis (IVCFT) is a common complication following filter deployment. The risk of IVCFT correlates positively with dwell time, with an overall reported incidence of approximately 32.58% (2). IVCFT is a frequent cause of failed retrieval attempts. Currently, advanced techniques such as loop snaring, laser sheath assistance, and endobronchial forceps are effective for addressing retrieval difficulties caused by filter tilt, fracture, or endothelial adhesion. However, the efficacy of these standard techniques is limited when dealing with significant thrombus burden within the filter. Consequently, there is an urgent need to develop safe and effective strategies specifically tailored for patients with IVCFT.

In this study, we propose an alternative technique that leverages the controllable deformation characteristics of spindle-shaped IVC filters (e.g., Illicium, Aegisy). By integrating this mechanical property with the retrieval process, we achieved an “en-bloc” removal of the filter-thrombus complex. This approach aims to address the technical challenges associated with retrieving filters containing high thrombus loads, offering a novel solution for clinical practice.

## Materials and methods

### Patient population

This single-center, retrospective, observational study was approved by the Institutional Review Board (IRB), and written informed consent was obtained from all participating patients. We retrospectively analyzed the clinical data of patients who underwent IVC filter retrieval utilizing the “filter-thrombus integrated retrieval technique.”

The inclusion criteria were as follows: (1) Cessation of the clinical indication for IVC filter retention; (2) Angiographic confirmation (via IVC venography) of a significant thrombus burden within or surrounding the filter, where standard snare retrieval was deemed to carry a high risk of pulmonary embolism (PE); (3) Intact filter structure without radiographic evidence of fracture; (4) Implantation of spindle-shaped IVC filters; (5) Presence of contraindications to anticoagulation/thrombolysis, or persistence of thrombus refractory to effective debulking therapies; and (6) Filter indwelling time exceeding the recommended retrieval window (extended dwell time).

The exclusion criteria were as follows: (1) Patients with symptomatic PE or radiographic evidence of PE on computed tomography pulmonary angiography (CTPA); (2) Contraindications to iodinated contrast media; and (3) Severe cardiac, hepatic, or renal insufficiency.

Anticoagulation Protocol For patients without contraindications to anticoagulation, Low Molecular Weight Heparin (LMWH) was administered subcutaneously at 100 AxaIU/kg twice daily (b.i.d.) during hospitalization following filter implantation. Post-discharge anticoagulation consisted of oral Rivaroxaban (15 mg b.i.d. for the first three weeks, followed by a maintenance dose of 20 mg once daily [q.d. ).

Baseline demographic and epidemiological characteristics of the patients are summarized in Table 1. Details regarding filter implantation and thrombus characteristics are presented in Table 2.

**Table 1:** Baseline characteristics of the study population.

Characteristics	Value (*N* = 51)
Gender, *n* (%)	
Male	29 (56.86%)
Female	22 (43.13%)
Age (years), mean ± SD	58.89 ± 11.38
Indications of IVCFs, *n* (%)	
DVT with contraindication to anticoagulation	15 (29.41%)
Major surgery	11 (21.57%)
High risk of bleeding	12 (23.53%)
Recurrent DVT	13 (25.49%)

**Table 2:** Thrombus volume, filter type, and dwelling time.

Variables	Value
Thrombus volume (cm^3^), mean ± SD	4.60 ± 0.42
Types of inferior vena cava filters, *n* (%)	
lllicium	27 (52.94%)
Aegisy	24 (47.06%)
Filters dwelling time (days), mean ± SD	35.25 ± 5.80

### Thrombus assessment

**Figure 1:**
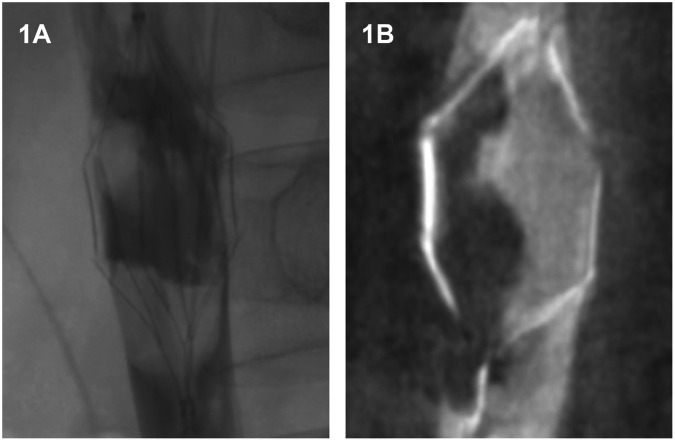
Assessment of thrombus burden. **(A)** Angiography confirming the presence of thrombus within the filter. **(B)** Reconstructed cross-sectional images derived from 3D volumetric acquisition of the IVC and filter, utilized for the delineation of the Region of Interest (ROI).

Thrombus burden was initially evaluated using Digital Subtraction Angiography (DSA). All image analyses were conducted on a dedicated DSA workstation. Upon angiographic confirmation of filter-associated thrombus (Figure 1A), a 3D data acquisition protocol was initiated to obtain volumetric data of the IVC. Each acquisition sequence consisted of two rotational runs: an initial non-contrast mask run, followed by a contrast-enhanced fill run performed during complete opacification of the region of interest. Patient immobilization was strictly maintained between scans to ensure subtraction accuracy.

The acquired raw rotational data were automatically transferred to a companion post-processing workstation utilizing integrated 3D reconstruction software. On the reconstructed cross-sectional images, the Region of Interest (ROI) measurement tool was employed to manually delineate the thrombus boundaries—defined as areas of contrast filling defects—in a slice-by-slice manner (Figure 1B). Thrombus identification relied on the significant density gradient between the thrombus and the normal contrast-opacified vascular lumen. The workstation software automatically calculated the voxel volume of the segmented regions and converted it into physical volume based on the angiographic calibration scale (Figure 1C).

To assess inter-observer reproducibility, 20% of the datasets were randomly selected for independent measurement by two radiologists with equivalent experience, and the Intraclass Correlation Coefficient (ICC) was calculated to quantify agreement.

### Filter-thrombus integrated retrieval technique

This technique leverages the inherent structural characteristics of spindle-shaped IVC filters to construct a “thrombus retrieval cage” using the apical filtration net and a portion of the struts, thereby achieving simultaneous thrombus clearance and filter retrieval.

The procedural steps were as follows: Venous access was established via the femoral vein, and a 12F long retrieval sheath was introduced. A snare was advanced over a guidewire to accurately capture the filter's retrieval hook. The snare was tightened, and the retrieval sheath was advanced to capture only the caudal anchoring barbs and the lower segment of the struts, thereby detaching them from the IVC wall. Crucially, the apical filtration net, the upper segment of the struts, and the entrapped thrombus were deliberately left unsheathed (exposed).

**Figure 2:**
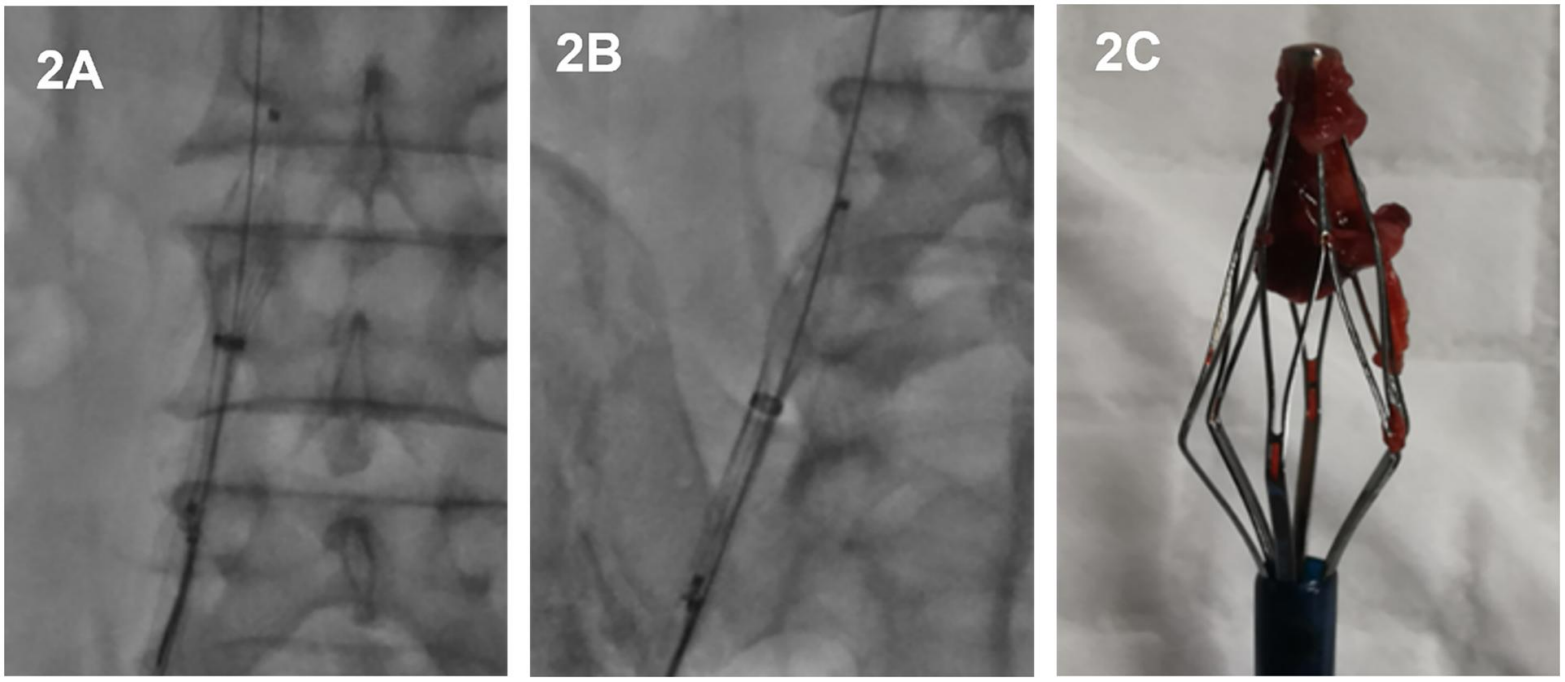
Illustration of the “filter-thrombus integrated retrieval” procedure. **(A)** After capturing the retrieval hook with a snare, the caudal anchoring barbs and partial struts are retracted into the sheath, while the apical filtration net remains unsheathed to entrap the thrombus. **(B)** The filter and the entrapped thrombus are slowly withdrawn *en bloc* through the venous tract as a single complex. **(C)** Gross photograph of the retrieved complex, showing the thrombus tightly encapsulated within the filter net.

Consequently, the filter transformed into a “diamond-shaped” configuration—tapered at both ends and expanded in the middle. This configuration effectively confined the thrombus within the basket, creating a stable “filter-thrombus complex” (Figure 2A). Under continuous fluoroscopic monitoring, the complex was slowly withdrawn distally *en bloc*, while strictly maintaining its “diamond-shaped” morphology (Figure 2B).

Upon the complex's arrival at the common femoral venotomy site, the patient was instructed to perform the Valsalva maneuver. The retrieval sheath and the filter complex were then simultaneously extracted through the access tract. Macroscopic examination *ex vivo* confirmed that the thrombus was tightly encapsulated within the retrieved filter (Figure 2C). Subsequently, iliac and IVC venography was performed via a dorsal foot vein to assess vascular morphology and screen for any potential injury to the iliac vein or IVC.

### Outcome measures

The primary objective of this study was to evaluate the safety and efficacy of the “filter-thrombus integrated retrieval technique” for the management of thrombosed IVC filters.

The primary endpoint was the technical success rate. Technical success was defined as the successful *en bloc* retrieval of the IVC filter together with the entrapped thrombus via the venous access, with post-procedural venography demonstrating patency of the IVC and iliac veins and the absence of significant residual thrombus or filter fragments.

The secondary endpoints included: (1) Incidence of PE: As a core metric for safety assessment, all patients underwent routine postoperative Computed Tomography Pulmonary Angiography (CTPA) to detect potential thrombus dislodgment into the pulmonary arterial system, regardless of the presence of clinical symptoms. (2) Procedure-related complications: These included femoral vein avulsion/laceration, access site hematoma, and arteriovenous fistula. 

## Results

A total of 51 patients were enrolled in this study, comprising 29 males and 22 females, with a mean age of 58.89 ± 11.38 years. The mean filter dwell time was 35.25 ± 5.80 days (range: 25–51 days). Inter-observer reproducibility for 3D thrombus volume measurement was good, with an Intraclass Correlation Coefficient (ICC) of 0.842.Pre-retrieval IVC venography confirmed varying degrees of thrombus burden within the filters in all patients; notably, 13 cases presented with severe thrombus burden (filling defect occupying >50% of the filter volume).

Utilizing the “filter-thrombus integrated retrieval technique,” the technical success rate was 100% (51/51). All filters, together with the entrapped thrombus, were successfully withdrawn via the venous tract and removed from the body as an intact complex. Post-procedural macroscopic examination confirmed that intact thrombus tissue was tightly encapsulated within the retrieved filter basket (Figure 2C).

Post-procedural venography demonstrated patent lumens of the IVC and iliac veins with good contrast filling. No angiographic evidence of vascular injury, such as contrast extravasation, dissection, or perforation, was observed.

Regarding safety, no cases of symptomatic PE were observed. Routine postoperative CTPA screening revealed signs of minor PE in 4 patients. Minor access site hematomas occurred in 6 patients (11.7%), all of which resolved with manual compression. No serious procedure-related complications occurred.

## Discussion

Retrievable IVC filters play a pivotal role in preventing fatal PE. However, guidelines from the Society of Interventional Radiology (SIR) recommend filter retrieval as soon as the risk of PE subsides. Previous studies have confirmed that prolonged indwelling time significantly increases the risk of complications such as migration, perforation, and fracture. Specifically, conical or “umbrella-shaped” filters (e.g., Denali, Celect) are predominantly associated with tilt and migration, whereas spindle-shaped IVC filters (e.g., OptEase) are more susceptible to IVC filter thrombosis (IVCFT), likely due to their distinct structural characteristics (3, 4). According to SIR guidelines, filter retrieval should be considered when filter-related complications occur.

Current literature (5, 6) suggests that standard retrieval techniques are safe for filters containing small thrombi (<1 × 1 cm). For larger thrombi (>1 × 1 cm), strategies such as extended anticoagulation or thrombus reduction via Catheter-Directed Thrombolysis (CDT) or Percutaneous Mechanical Thrombectomy (PMT) are recommended to reduce thrombus volume (7–10). However, in clinical practice, physicians inevitably encounter patients who are refractory to thrombus reduction therapies or have absolute contraindications to anticoagulation/thrombolysis. Furthermore, extended dwell times often lead to endothelialization and fibrotic encapsulation of the filter legs, further complicating retrieval.

In cases of high thrombus burden, attempting standard retrieval—defined as retracting the filter completely into the sheath—carries a high risk of iatrogenic PE. The underlying mechanism involves the centripetal contraction of the filter struts as the snare is tightened and the sheath advanced. This mechanical action exerts a strong squeezing effect on the entrapped or *in situ* thrombus, potentially causing fragmentation and downstream migration of emboli into the pulmonary arteries.

To mitigate this risk, some authors (11) have proposed the prophylactic placement of a second filter in the suprarenal IVC to serve as a “temporary embolic protection net.” Although this strategy can effectively intercept dislodged emboli, our center's experience suggests several limitations: (1) The use of an additional filter and associated consumables significantly escalates healthcare costs; (2) If the protective filter captures a significant amount of debris, it instantly transforms into a new “thrombosed filter.” This not only potentially impedes renal venous return but also poses the identical retrieval challenges associated with the original device; (3) Repeated filter deployment and manipulation increase the cumulative risk of mechanical injury to the IVC wall.

This study evaluated the “filter-thrombus integrated retrieval technique” in 51 patients with complex IVC filter thrombosis. High technical success was achieved even in cases with severe thrombus burden (>50% filter volume), with no occurrences of symptomatic PE or major venous injury.

A critical safety consideration in this study pertains to the management of the venous access site. Given that the technique involves extracting the expanded filter-thrombus complex directly through the common femoral venotomy, there exists a theoretical risk of venous avulsion or significant hemorrhage. However, our complication data revealed no instances of femoral vein avulsion or arteriovenous fistula among the 51 patients. Only minor access site hematomas or oozing were observed, all of which were successfully managed via routine manual compression.

We recommend that in cases presenting with palpable resistance during extraction, the operator should perform a limited dermatotomy or blunt dissection of the subcutaneous track. This maneuver not only mitigates vascular injury caused by excessive traction but also prevents the “stripping” or dislodgment of the entrapped thrombus caused by tissue compression as the filter net traverses the skin.

The core mechanism underlying this technique's safety lies in the controlled deformation of the filter to create a “diamond-shaped retrieval cage.” Unlike standard techniques that collapse the filter completely—potentially squeezing the thrombus out—our approach selectively retrieves the caudal anchors while maintaining the apical filtration net in an expanded state (Figure 2). This configuration functions as a “closed compartment” that physically entraps the thrombus during withdrawal. The gross examination of retrieved specimens, which showed thrombus tightly encapsulated within the filter mesh, empirically validates the efficacy of this “caging” effect.

Furthermore, by extracting the complex *en bloc*, this strategy offers a “one-stop” solution that may obviate the need for adjunctive thrombolysis or costly aspiration devices, particularly in patients with contraindications to lytics. Although minor asymptomatic PE was detected in 7.8% of patients, these resolved with anticoagulation alone, suggesting that the technique effectively prevents clinically significant embolization.

## Limitations

This study has several limitations. First, it was a single-center, retrospective study with a relatively small sample size (*n* = 51), which may introduce selection bias. Second, the study lacked a concurrent control group (e.g., a routine thrombolysis group); thus, future Randomized Controlled Trials (RCTs) with larger cohorts are warranted to further validate its comparative efficacy against conventional treatments.

Third, the sensitivity of CTPA for detecting minute thrombi at the sub-segmental or microvascular level is limited. Consequently, the 7.8% (4/51) incidence of asymptomatic PE reported in this study may potentially underestimate the actual rate of micro-embolic events.

Fourth, the assessment of IVC wall injury relied primarily on IVC venography, which possesses significantly lower sensitivity than Intravascular Ultrasound (IVUS) in identifying minor intimal injuries or non-transmural vessel wall damage. Therefore, we cannot entirely exclude the possibility that clinically silent intimal abrasions or micro-tears, which were angiographically occult, may have occurred during filter disengagement. Finally, this study focused primarily on perioperative outcomes, and long-term follow-up data regarding venous patency are currently lacking.

## Conclusion

In summary, our findings suggest that the “filter-thrombus integrated retrieval technique,” which leverages the distinct structural characteristics of spindle-shaped filters, is a safe and cost-efficient strategy for the management of thrombosed IVC filters. By mitigating the risk of iatrogenic pulmonary embolism via a unique physical mechanism, this technique offers a novel therapeutic paradigm for the clinical management of such complex cases.

## Data Availability

The raw data supporting the conclusions of this article will be made available by the authors, without undue reservation.
